# Suture length to wound length ratio in 175 small animal abdominal midline closures

**DOI:** 10.1371/journal.pone.0216943

**Published:** 2019-05-20

**Authors:** Verena Winter, Brigitte Degasperi, Barbara Bockstahler, Gilles Dupré

**Affiliations:** Department for Small Animals and Horses, Division of Small Animal Surgery, University of Veterinary Medicine, Vienna, Austria; University of Bari, ITALY

## Abstract

Experimental and human studies have reported the advantages of a suture length to wound length (SL:WL) ratio greater than 4:1 in midline abdominal closure. This is achieved when the tissue bite (TB) is equal to or larger than the stitch interval (SI). Although TB and SI values are recommended in some textbooks, SL:WL ratios are rarely reported in veterinary textbooks. Additionally, no clinical data regarding these parameters could be found in small animals. Therefore, the aim of this study was to evaluate the SL:WL ratio of midline laparotomy closure in dogs and cats performed by surgeons with different levels of expertise and to compare the findings with current textbook recommendations. Midline laparotomy incisions of 100 dogs and 75 cats were closed in continuous pattern by diplomates and residents of both the European College of Veterinary Surgeons (ECVS) and the European College of Animal Reproduction (ECAR). The mean SL:WL ratio was 2.5 ± 0.7:1. The surgeons´ level of experience and the species and body weights of the animals did not have any significant influence on the SL:WL ratio. A moderate negative correlation was observed between the mean SI to mean TB (SI:TB) ratio and the SL:WL ratio. In this study, the mean SI matched the textbook recommendations both in feline and canine species, whereas the TB in cats was different. In this study, the SL:WL ratio was less than 4:1 without apparent complications. Because of the low prevalence of incisional hernia in dogs and cats larger studies are necessary to evaluate clinical significance of the presented data.

## Introduction

In human surgery, a ventral midline incision is a frequently used approach to the abdominal cavity [1,2]. Because complications of abdominal closure generate pain and morbidity and cause a substantial financial burden to the healthcare system, experimental and clinical trials have been conducted to reduce the risk of incisional hernias [3–6]. In humans, the incidence of incisional hernia can be as high as 18% depending on closure techniques [4,6–10]. In high-risk patients, the five-year incidence of incisional hernia after vascular repair of abdominal aortic occlusive and aneurysmal disease is 69.1% [11]. With this risk in mind, recommendations for abdominal incision closure have been revised in human surgery. For instance, the European Hernia Society recommends that elective midline abdominal incisions be closed with a continuous pattern including the aponeurotic tissue utilizing slowly absorbable, monofilament suture material with small tissue bites (TBs) and a suture length to wound length (SL:WL) ratio of at least 4:1 [2]. The SL:WL ratio is defined as the quotient of the length of the suture (SL) being used to the WL [12]. Studies have demonstrated that the rate of incisional hernia is almost three times higher if the midline incision is sutured with an SL:WL ratio less than 4:1 [9,10,12–14].

Lastly, some studies have reported that the SL:WL ratio acts as a tool to monitor the quality of the surgeons’ suture technique and that standardization of ventral midline abdominal closure could possibly be achieved by this tool [1,13,14].

Although in veterinary medicine a ventral midline incision is the most frequently used approach to the abdomen [15–17], we are not aware of any clinical studies evaluating the SL:WL ratio in closure of abdominal incisions in small animals. In horses, one cadaver study evaluated the influence of two different stitch intervals (SIs) (1.5 cm and 1.0 cm) with a TB of 1.5 cm in a continuous pattern used to close a ventral midline incision on the SL:WL ratio. The mean SL:WL ratios resulting in a 4.5 ± 0.7 and 4.8 ± 0.8, respectively [18]. No significant difference between the SL:WL ratio could be identified.

Klonner [19] investigated the SL:WL ratio in an in vitro abdominal closure setup performed by three groups of surgeons with different levels of experience (non-experienced, moderately-experienced and highly-experienced). In that study, the mean TB was 5.5 mm and the mean SI was 5.2 mm. The mean SL:WL ratio of all sutures was 4.1:1, with 60% of all sutures being above 4:1. Furthermore, no significant difference among the three groups was observed, and a significant correlation between the SL:WL ratio and the SI to TB (SI:TB) ratio was found.

In current small animal veterinary textbooks, different recommendations are given for TBs and SIs, as shown in Table 1.

**Table 1:** Overview of textbook recommendations.

Textbook	TB [mm]	SI [mm]
Shales C. [20]	3–5	3–5
Rosin E. [21]	3–10	5–10
Fossum TW. [22]	4–10	5–10
Smeak D. [23]	5–7	3–4
Tobias KM. [24]	5–10	5–10
Bellenger CR. [16]	5–10	3–12

TB = tissue bite, SI = stitch interval.

The suggestion to use TBs and SIs depending on the animal´s size is common in all textbooks [16,21–24], but no recommendation regarding the SI:TB ratio and stitch length (STL) was found in any textbook. Likewise, we are not aware of any clinical study evaluating the TB, SI, SI:TB ratio or SL:WL ratio in small animal abdominal closure.

The present study was designed to evaluate the SL:WL ratio in abdominal midline closure in small animals in a clinical setup. We hypothesized that veterinary surgeons would use an SL:WL ratio above 4:1, that surgical experience would influence the SL:WL ratio, that the TB and SI would match the textbook recommendations and that patients with Body Condition Score (BCS) higher than 5 would be sutured with significantly higher SL:WL ratios than patients with ideal BCS.

## Materials and methods

The study design was approved by the Institutional Ethics and Animal Welfare Committee of the University of Veterinary Medicine, Vienna and the Ethics Committee of the Medical University of Vienna in accordance with Good Scientific Practice guidelines and national legislation (Approval No. ETK 13/09/2016). Participation was voluntary, and the owners provided written consent to be enrolled.

### Test groups

For evaluation of surgeon´s experience two groups, each consisting of five surgeons, were generated: Group Diplomates included diplomates of the ECVS and ECAR, Group Residents included residents of the ECVS and ECAR. To evaluate the minimum number of cases for each participant to reach the margin of interest with an SL:WL ratio between 4:1 and 5:1, power calculations were based on the results of Klonner’s study [19]. The statistical power analysis program G*Power v3.1 was used and a minimum power of 80% with an alpha error of 5% was accepted. Prior to the study, the surgeons were verbally informed of the evaluation of their closure technique. Detailed instructions were given on how to measure the SL and WL.

### Animals

#### Inclusion criteria

The study took place at the small animal surgery clinic of the University of Veterinary Medicine, Vienna between November 2016 and January 2018.

All animals with owner consent that underwent midline celiotomy performed by a surgeon enrolled in the study were included.

### Surgery

A slowly absorbable monofilament suture composed of synthetic polyester (Glycomer 631 Biosyn Covidien Austria GmbH, Brunn am Gebirge, Austria) with a size ranging from 3–0 to 1 United States Pharmacopeia (USP) based on the patient’s size and surgeon’s preference was used for the closure. The memory of the suture material was released by gentle traction before closure was started. All ten participating surgeons were right handed and sutured from right to left. The surgeons sutured the external sheath of the rectus abdominis muscle in a continuous pattern. The closure was accomplished with just enough tension on the tissue and/or on suture material to achieve appositional closure. The first knot (7 throws) and the final knot (8 throws) were placed just outside the incision line. A sterile, single-use paper measuring tape (cm/mm) (production company: IKEA, Stichting INGKA Foundation, Delft) was used to measure the WL and SL.

### Outcome parameters

#### Suture parameters

**Fig 1:**
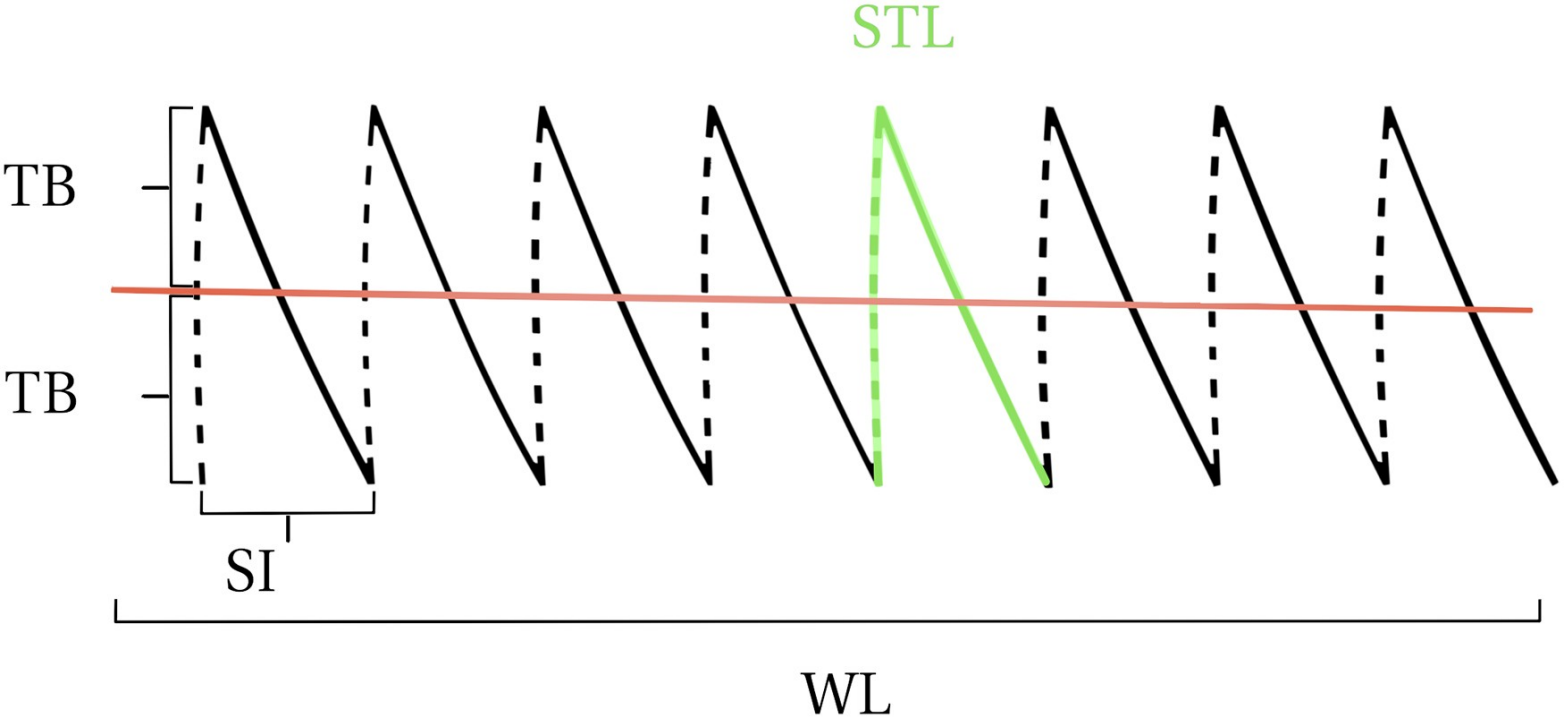
Abdominal midline laparotomy closure. TB = tissue bite, STL = stitch length, SI = stitch interval, WL = wound length.

1. The SL [mm] was calculated by measuring the total SL after positioning the first knot and remaining SL before positioning the last knot. Subtraction of the remaining SL from the total SL yielded the SL.
2. The WL [mm] was measured after closure.
3. Number of stitches: A video clip recording was obtained for each procedure (Sony Cyber-shot DSC-RX 100/Panasonic Lumix DMC-LX7), and the number of stitches was counted by analysis of each video clip.
4. The mean SI [mm] was calculated by dividing the WL [mm] by the number of stitches.
5. The mean STL [mm] was calculated by dividing the SL [mm] by the number of stitches.
6. The mean TB [mm] was calculated with the Pythagorean theorem using the following formula:
  $$TB=\frac{{STL}^{2}{-SI}^{2}}{4STL}$$
  where TB = tissue bite, STL = stitch length, SI = stitch interval.
  The Pythagorean theorem applied to abdominal closure is shown in Fig 1 and the mathematical approach is shown in S1 Data.
7. For some descriptive statistical analyses, the SL:WL ratio was categorized as <2:1, 2:1-<3:1, 3:1-<4:1 and 4:1-<5:1.
8. The mean stitch interval to mean tissue bite (SI:TB) ratio was calculated by dividing the mean SI by the mean TB.
9. Body weight [kg]
10. The nine-point BCS system was used for evaluation, and a BCS of 5 was defined as ideal [25–26].

### Follow-up

Routine follow-up examination was planned 10 to 14 days after surgery for suture removal and clinical evaluation for possible hernia formation. Reevaluation of the abdominal wall was planned for 2 to 6 months later whenever possible.

### Statistical analysis

The statistical analysis was performed using IBM SPSS v24. Descriptive statistics were compiled for SL:WL ratios in dogs and cats including the mean TB ± standard deviation (SD), mean STL ± SD and mean SI ± SD divided by group Diplomates, group Residents and species, minimum and maximum range of the SL:WL ratio, mean SL:WL ratio ± SD, SL:WL ratio classifications and number of sutures. Further descriptive statistics were used to describe mean stitch interval and mean tissue bite according to weight categories. A general linear model was used to evaluate the impact of species and group Diplomates versus group Residents on the SL:WL ratio controlling for body weight (ANOVA). Furthermore, Pearson’s correlation coefficients between the SL:WL ratio and outcome parameters were calculated. The assumption of a normal distribution was tested using the Kolmogorov-Smirnov test. Statistical significance was accepted at p<0.05. Data are described as the mean ± SD.

## Results

In total, 100 dogs and 75 cats were studied. Within each species, each surgeon performed 5 to 13 closures. The mean age of the 175 animals was 5.9 ± 4.4 years (range, 2 months to 17 years), the mean body weight in dogs was 19.0 ± 13.8 kg (range, 2 to 75 kg), and the mean body weight in cats was 3.7 ± 1.1 kg (range, 2.1 to 8 kg). There were 118 females, 36 spayed females, 57 males and 27 neutered males. The mean BCS was 5 (range, 2 to 8) in 49.7% of the animals. Descriptive data of the animals are displayed in S1 Table. An overview of the breeds is given in S2 Table. The types of surgical procedures performed are listed in S3 Table.

### Suture length to wound length ratio

The overall mean wound length was 9.7 ± 5.6 cm (range, 1.6 to 26.0 cm) and the overall mean suture length was 24.6 ± 16.1 cm (range, 1.9 to 68.1 cm). The overall range of the SL:WL ratio varied from 1.1:1 to 4.5:1, as shown in Table 2, with an overall mean SL:WL ratio ± SD of 2.5 ± 0.7:1. The majority of the closures (n = 78, 44.6%) were within a ratio of 2:1-<3:1, as reflected in Table 3. Only 5/175 (2.9%) closures were above 4:1. When the SL:WL ratio from the group Diplomates was compared to that of the group Residents no statistical difference was detected (2.4 ± 0.7:1 versus 2.5 ± 0.7:1, p = 0.302). The group Diplomates used a ratio of 1.2 to 4.5:1 (2.4 ± 0.7:1), and the group Residents used a ratio of 1.1 to 4.3:1 (2.5 ± 0.7:1), as shown in Table 2. The majority of the sutures performed by the group Diplomates (41.1%) and the majority of sutures performed by the group Residents (48.8%) showed a ratio of 2:1-<3:1, as shown in Table 3.

**Table 2:** Minimum and maximum ranges of the SL:WL ratio and mean SL:WL ratio ± SD separated by groups and species.

Group	Species	n	Minimum	Maximum	Mean SL:WL ratio ± SD
Diplomates	dog	53	1.2	3.7	2.5 ± 0.7
Residents		47	1.2	4.3	2.5 ± 0.6
Diplomates	cat	42	1.2	4.5	2.4 ± 0.8
Residents		33	1.1	4.2	2.6 ± 0.9

SL:WL ratio = suture length to wound length ratio, SD = standard deviation, n = number of sutures.

**Table 3:** SL:WL ratio classifications and number of sutures per classification performed by group Diplomates and group Residents in dogs and cats.

SL:WL ratio	Sutures			Dogs		Cats	
	Total sutures	Dipl.	Residents	Dipl.	Residents	Dipl.	Residents
<2:1	49	30	19	13	12	17	7
	28.0%	31.6%	23.8%	24.5%	25.5%	40.5%	21.2%
2:1-<3:1	78	39	39	24	24	15	15
	44.6%	41.1%	48.8%	45.3%	51.1%	35.7%	45.5%
3:1-<4:1	43	24	19	16	10	8	9
	24.6%	25.3%	23.8%	30.2%	21.3%	19.1%	27.3%
4:1-<5:1	5	2	3	0	1	2	2
	2.9%	2.1%	3.8%	0.0%	2.1%	4.8%	6.1%

SL:WL ratio = suture length to wound length ratio, Dipl. = Diplomates.

The results divided by species revealed the following: the group Diplomates closed the abdominal incisions in dogs with a mean SL:WL ratio of 2.5 ± 0.7:1 and in cats with a mean SL:WL ratio of 2.4 ± 0.8:1. The group Residents closed with a mean SL:WL ratio of 2.5 ± 0.6:1 in dogs and with a mean SL:WL ratio of 2.6 ± 0.9:1 in cats. The ratios are presented in Table 2. The statistical analysis did not reveal any influence of the species (p = 0.955) and did not show an association between species and groups (p = 0.233). Furthermore, patients’ body weights did not have a significant influence on the SL:WL ratio (p = 0.996).

#### Correlation between the SL:WL ratio and the suture parameters, the mean SI:TB ratio and the morphometric data

Descriptive statistics and correlation coefficients for the mean suture parameters, mean SI:TB ratio and morphometric data are shown in Table 4. All correlations are described according to the study by Mukaka et al. [27]. A moderate negative correlation was found between the mean SI:TB ratio and the SL:WL ratio. Moderate positive correlations were found between the SL:WL ratio and the mean TB, mean STL and SL. A low positive correlation was found between the number of stitches and the SL:WL ratio. A negligible correlation was found between the WL and the SL:WL ratio. The mean SI, body weight, BCS and suture size showed no correlations with the SL:WL ratio.

**Table 4:** Correlation between the SL:WL ratio and different parameters.

Evaluated parameters	Mean ± SD	Correlation to SL:WL ratio	
		r	p
Suture size	NA	0.09	0.242
Suture length [mm]	246.2 ± 160.6	0.52	<0.001
Wound length [mm]	96.6 ± 56.3	0.16	<0.041
Number of stitches	17.1 ± 6.6	0.31	<0.001
Stitch interval [mm]	5.4 ± 2.1	0.02	0.766
Stitch length [mm]	13.4 ± 6.3	0.57	<0.001
Tissue bite [mm]	3.0 ± 1.6	0.68	<0.001
Stitch interval to tissue bite ratio	2.2 ± 1.3	-0.54	<0.001
Body weight [kg]	12.4 ± 12.9	-0.02	0.822
BCS (1–9)	NA	0.05	0.501

SL:WL ratio = suture length to wound length ratio, SD = standard deviation, r = Pearson correlation coefficient, p = probability value, NA = not applicable.

#### Influence of groups and species on SL:WL ratio, suture parameters, mean SI:TB ratio, and morphometric data

Descriptive statistics and correlation coefficients divided by species and groups for the suture parameters, mean SI:TB ratio and morphometric data are presented in Table 5. Higher correlations were found in the group Diplomates suturing cats than in the other groups (namely, diplomates suturing dogs and residents suturing both dogs and cats). High negative correlations between the SL:WL ratio and mean SI:TB ratio were found in dogs and cats performed by the group Diplomates and in dogs performed by the group Residents. Also high negative correlations between the SL:WL ratio and mean TB were found in dogs and cats performed by the group Residents and in cats performed by the group Diplomates. The mean SI and BCS showed no correlations.

**Table 5:** Correlation between the SL:WL ratio and different parameters separated by groups and species.

Evaluated parameters	Correlation to the SL:WL ratio											
	Diplomates						Residents					
	Dogs			Cats			Dogs			Cats		
	*Mean* ± *SD*	*r*	*p*	*Mean* ± *SD*	*r*	*p*	*Mean ± SD*	r	*p*	*Mean* ± *SD*	*r*	*p*
Suture size	NA	-0.98	0.487	NA	0.25	0.095	NA	0.34	0.026	NA	-0.03	0.861
Suture length [mm]	*321*.*6* ± *163*.*3*	0.52	<0.001	172.2 ± *136*.*2*	0.82	<0.001	*277*.*0* ± *153*.*5*	0.47	0.001	*175*.*5* ± *127*.*4*	0.49	0.004
Wound length [mm]	*128*.*5* ± *56*.*4*	0.03	0.830	*66*.*2* ± *38*.*0*	0.52	<0.001	*110*.*9* ± *55*.*0*	0.07	0.656	*63*.*9* ± *39*.*7*	0.25	0.163
Number of stitches	*20*.*6* ± *6*.*0*	0.19	0.170	*14*.*2* ± *5*.*5*	0.7	<0.001	*17*.*6* ± *6*.*2*	0.13	0.393	*14*.*7* ± *6*.*6*	0.36	0.041
Stitch interval [mm]	*6*.*2* ± *2*.*2*	-0.07	0.645	*4*.*4* ± *1*.*6*	0.22	0.172	*6*.*2* ± *2*.*0*	-0.02	0.916	*4*.*0* ± 1.3	0.04	0.842
Stitch length [mm]	*15*.*5* ± *6*.*6*	0.55	<0.001	*10*.*7* ± *5*.*7*	0.77	<0.001	*15*.*3* ± *6*.*1*	0.59	<0.001	*10*.*6* ± *4*.*5*	0.63	<0.001
Tissue bite [mm]	*3*.*5* ± *1*.*6*	0.68	<0.001	*2*.*4* ± *1*.*4*	0.84	<0.001	*3*.*5* ± *1*.*5*	0.72	<0.001	*2*.*4* ± *1*.*2*	0.72	<0.001
Stitch interval to tissue bite ratio	2.1 ± *1*.*0*	-0.77	<0.001	*2*.*3* ± *1*.*1*	-0.74	<0.001	*2*.*0* ± *0*.*8*	-0.71	<0.001	*2*.*3* ± *2*.*1*	-0.54	<0.001
Body weight [kg]	*17*.*6* ± *11*.*4*	-0.31	0.024	*3*.*6* ± *0*.*9*	0.11	0.499	*20*.*3* ± *16*.*2*	0.17	0.263	*3*.*9* ± *1*.*3*	0.24	0.184
BCS (1–9)	*NA*	0.12	0.398	*NA*	0.15	0.346	*NA*	0.05	0.740	*NA*	-0.15	0.405

SL:WL ratio = suture length to wound length ratio, SD = standard deviation, r = Pearson correlation coefficient, p = probability value, NA = not applicable.

### Mean stitch interval, mean tissue bite and mean stitch length

**Fig 2:**
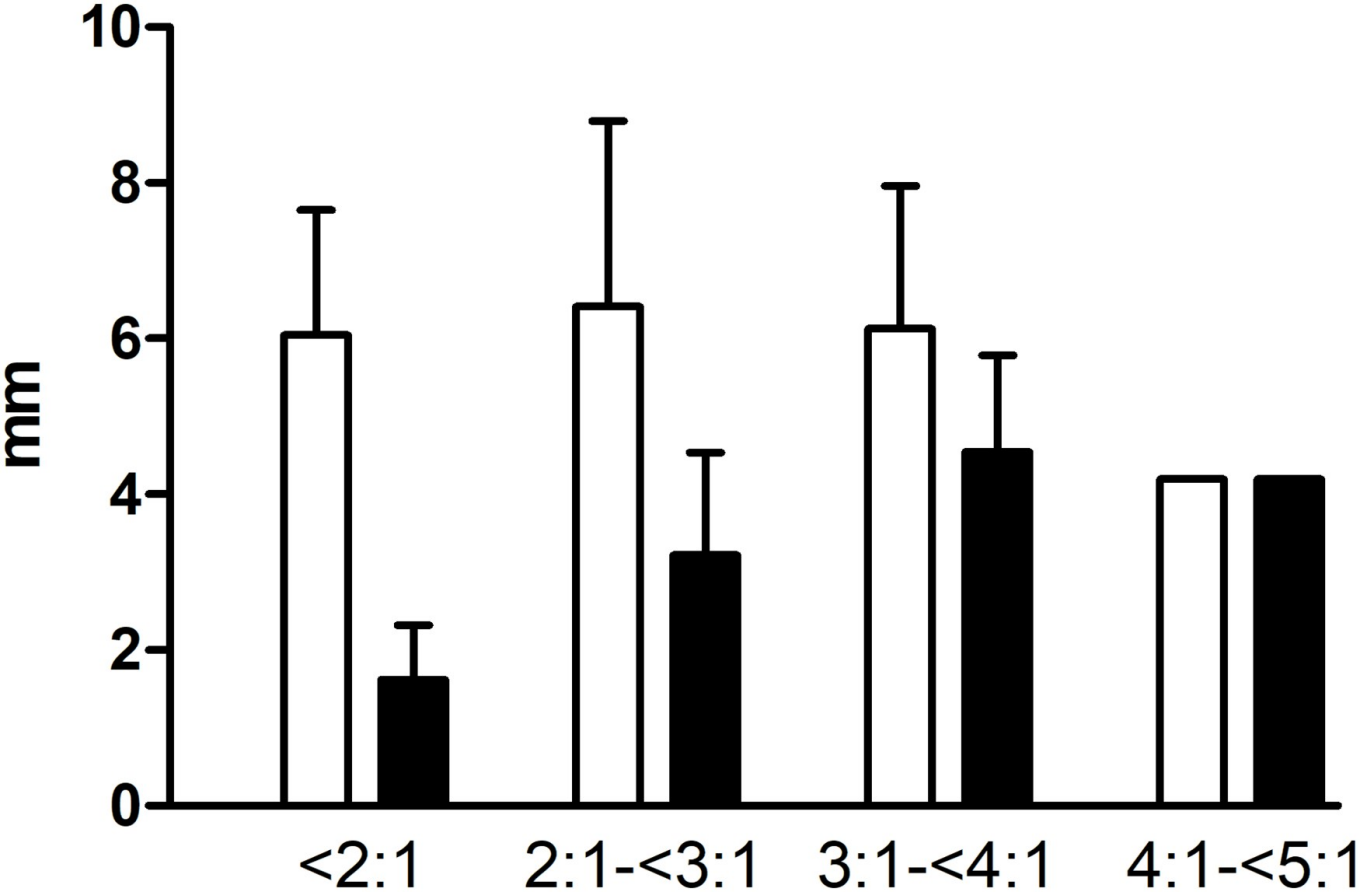
100 abdominal midline closure measurements in dogs categorized by SL:WL ratios <2:1, 2:1-<3:1, 3:1-<4:1, and 4:1-<5:1 analyzed for the mean SI ± SD and mean TB ± SD.

**Fig 3:**
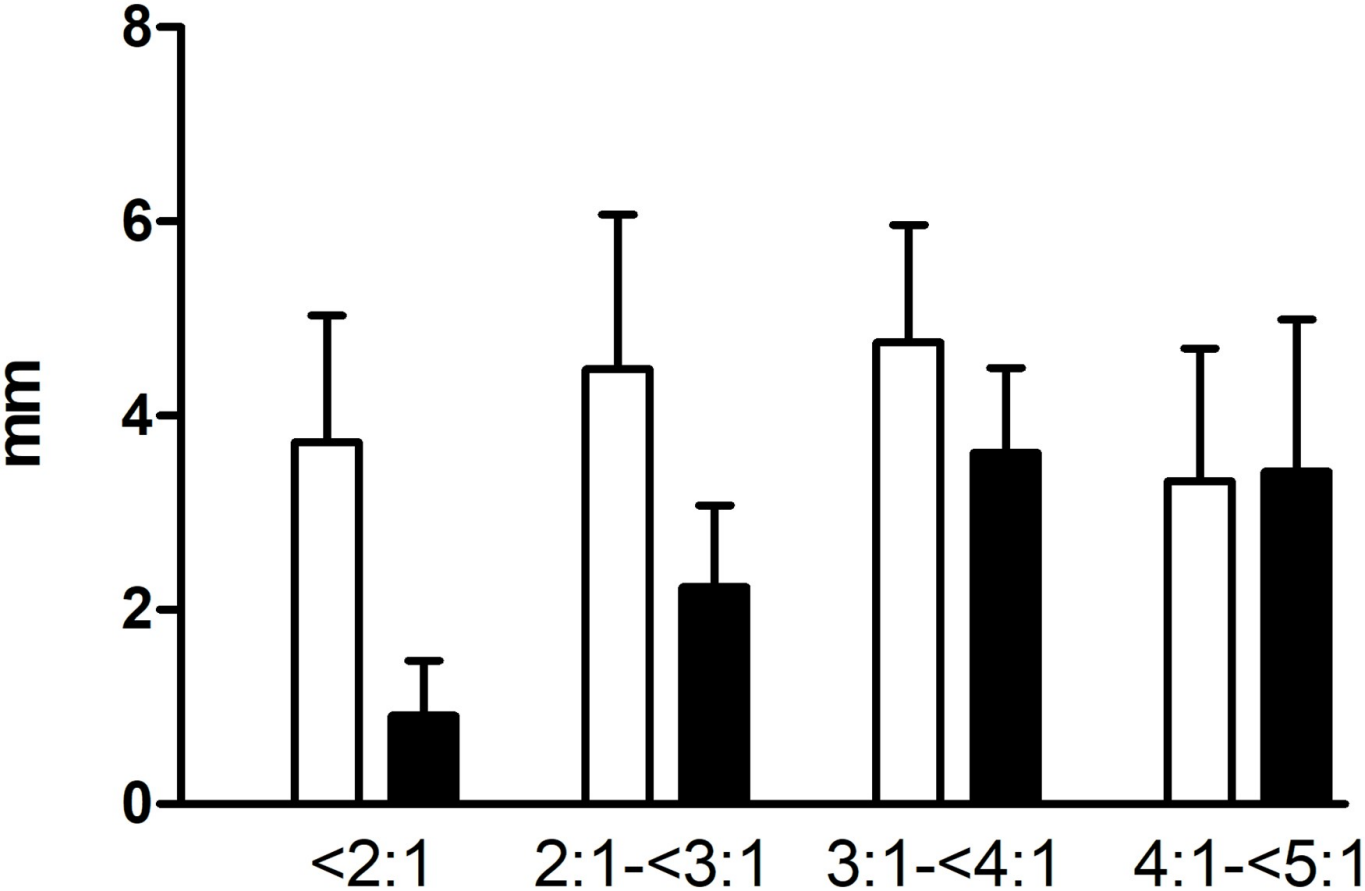
75 abdominal midline closure measurements in cats categorized by SL:WL ratios <2:1, 2:1-<3:1, 3:1-<4:1, and 4:1-<5:1 analyzed for the mean SI ± SD and mean TB ± SD.

The SL:WL ratios are presented in Table 6 with the mean SI ± SD, mean TB ± SD and mean STL ± SD. Most of the SL:WL ratios were less than 4:1. In these patients, the increase in the SL:WL ratio was due to an increase in the mean TB, mean STL and mean SI. It must be noted, however, that the mean SI remained nearly stable in dogs. The mean SI ± SD and TB ± SD categorized as SL:WL ratios <2:1, 2:1-<3:1, 3:1-<4:1 and 4:1-<5:1 are shown in Fig 2 for dogs and in Fig 3 for cats. Since not enough SL:WL ratios above 4:1 were obtained, the parameters that led to this value could not be objectively evaluated.

**Table 6:** Mean values and SDs for SI, TB and STL in dogs and cats classified by SL:WL ratios.

SL:WL ratio	Dogs				Cats			
	n	Mean SI ± SD [mm]	Mean TB ± SD [mm]	Mean STL ± SD [mm]	n	Mean SI ± SD [mm]	Mean TB ± SD [mm]	Mean STL ± SD [mm]
<2:1	25	6.1 ± 1.6	1.6 ± 0.7	10.2 ± 3.1	24	3.7 ± 1.3	0.9 ± 0.6	6.0 ± 2.7
2:1-<3:1	48	6.4 ± 2.4	3.2 ± 1.3	15.6 ± 6.1	30	4.5 ± 1.6	2.2 ± 0.8	10.8 ± 3.9
3:1-<4:1	26	6.1 ± 1.8	4.6 ± 1.2	20.1 ± 5.5	17	4.8 ± 1.2	3.6 ± 0.9	15.9 ± 3.8
4:1-<5:1	1	4.2	4.2	17.9	4	3.3 ± 1.4	3.4 ± 1.6	14.5 ± 6.5

n = number of sutures, Mean SI = mean stitch interval, Mean TB = mean tissue bite, Mean STL = mean stitch length, SD = standard deviation, SL:WL ratio = suture length to wound length ratio.

The mean stitch interval (SI) ± standard deviation (SD) [mm] is depicted in white, and the mean tissue bite (TB) ± SD [mm] is shown in black. Note that for a suture length to wound length (SL:WL) ratio of 4:1-<5:1, there was only one patient.

The mean stitch interval (SI) ± standard deviation (SD) [mm] is depicted in white, and the mean tissue bite (TB) ± SD [mm] is shown in black. Note that for a suture length to wound length (SL:WL) ratio of 4:1-<5:1, there were only four patients.

#### Comparison of the mean tissue bite and mean stitch interval with textbook recommendations

In dogs, the overall mean TB was 3.2 ± 1.6 mm, and the mean SI was 6.2 ± 2.1 mm. The mean TB corresponded with the range as defined in Rosin`s statements [21] but differed from the suggestions of Tobias [24], Fossum [22], Smeak [23] and Bellenger [16]. The mean SI coincided with the current textbook recommendations [16,21,22,24], but differed from the suggestions of Smeak [23].

In cats, the overall mean TB was 2.2 ± 1.3 mm, and the mean SI was 4.2 ± 1.5 mm. These results differ from the current veterinary textbook recommendations, where the minimum recommended TB is 3 mm. The mean SI was in the range of the recommendations [16,20].

#### Mean stitch interval, mean tissue bite for weight categories

The mean stitch interval ± standard deviation and the mean tissue bite ± standard deviation for weight categories are shown in Table 7 for dogs and Table 8 for cats.

**Table 7:** Mean values and SDs for SI and TB in dogs classified by weight [kg] and BCS.

Weight [kg]	n	Mean SI ± SD [mm]	Mean TB ± SD [mm]	BCS	n	Mean SI ± SD [mm]	Mean TB ± SD [mm]
<5	16	4.2 ± 1.3	2.4 ± 1.3	3	5	5.6 ± 0.9	3.0 ± 0.8
<10	18	4.9 ± 1.3	2.1 ± 1.1	4	18	6.5 ± 1.7	2.9 ± 1.6
10–20	23	6.1 ± 1.8	3.2 ± 1.6	5	50	5.7 ± 2.3	3.1 ± 1.6
20–30	21	7.3 ± 1.7	3.7 ± 1.6	6	22	6.8 ± 1.9	3.4 ± 1.5
>30	22	7.8 ± 1.6	4.0 ± 1.3	7	4	7.8 ± 4.8	3.7 ± 0.5
				8	1	8.4	6.4

Mean TB = mean tissue bite, Mean SI = mean stitch interval, n = number of sutures, SD = standard deviation, BCS = body condition score.

**Table 8:** Mean values and SDs for SI and TB in cats classified by weight [kg] and BCS.

Weight [kg]	n	Mean SI ± SD [mm]	Mean TB ± SD [mm]	BCS	n	Mean SI ± SD [mm]	Mean TB ± SD [mm]
<3	18	3.8 ± 1.5	1.7 ± 0.9	2	1	7.20	3.10
<4	36	4.2 ± 1.5	2.2 ± 1.4	3	6	5.5 ± 0.9	2.7 ± 0.6
<5	14	4.6 ± 1.2	2.1 ± 1.2	4	19	4.5 ± 1.7	2.3 ± 1.3
>5	7	5.6 ± 1.1	3.6 ± 1.0	5	37	3.6 ± 1.2	1.9 ± 1.4
				6	11	4.7 ± 1.1	2.6 ± 1.5
				7	1	6.4	2.9

Mean TB = mean tissue bite, Mean SI = mean stitch interval, n = number of sutures, SD = standard deviation, BCS = body condition score.

A moderate positive correlation was found between weight and mean SI (r = 0.642, p = <0.001) and a low positive correlation was found between weight and mean TB (r = 0.428).

A negligible correlation was found between weight [kg] and BCS (r = 0.282, p = <0.001). The mean TB and mean SI showed no correlation with the BCS.

### Follow-up

Twenty-nine (16.6%) animals met the follow-up requirements and were included in the follow-up. These animals had no incisional hernia on both examinations, 10–14 days after surgery and 2–6 months later. One patient showed a mild surgical site infection two days after surgery and was treated conservatively.

## Discussion

In our study, the majority of the participants applied an SL:WL ratio less than 4:1. Almost half of all sutures (44.6%) were within a ratio of 2:1-<3:1 with an overall mean SL:WL ratio of 2.5 ± 0.7:1. In comparison, in the experimental in vitro study performed at the same institution evaluating three different groups of surgeons (non-experienced, moderately-experienced and highly-experienced), 60.5% of all sutures yielded an SL:WL ratio above 4:1, and the mean SL:WL ratio was 4.1:1 [19]. In a recent study by Williams et al. [28] where surgeons were explicitly required to achieve an SL:WL ratio of 4:1, 76% of them did achieve a SL:WL ratio of ≥4:1. In contrast, in the present study, only 2.9% or five out of 175 incisions (one dog and four cats) were closed with an SL:WL ratio above 4:1.

There might be several reasons for these differences. Klonner [19] and Hope et al. [29] mentioned that an abdominal wall model could never truly imitate a real abdominal wall. Whether the relative stiffness of the model or whether SIs and TBs can be readily appreciated on the model compared to the true abdominal wall play a role is unknown. Israelsson [14] mentioned that introducing new suture material could influence the suture technique of surgeons and hence the calculated SL:WL ratio of the observed participants. In this clinical study, the slowly absorbable synthetic suture material Biosyn [30] was chosen as it had been in use for many years at the small animal surgery unit and thus was already known by the participants and should not have influenced the SL:WL ratio. In a study by Naleway et al.[31], Biosyn had the highest failure load of knotted and unknotted absorbable suture materials.

According to our results, expertise did not influence the SL:WL ratio. Diplomates and residents performed with a mean SL:WL ratio ranging between 2.4 ± 0.8:1 and 2.6 ± 0.9:1, respectively. In comparison, human medicine senior surgeons with more than 10 years of experience sutured wounds with a lower SL:WL ratio than junior surgeons in one study [13].

Morphometric data, such as BCS, did not correlate with the SL:WL ratio, and body weight showed no significant influence on the SL:WL ratio. This stands in contrast to human reports in which overweight people were closed with a significantly higher SL:WL ratio [32]. Furthermore, a high body mass index is reported to be a risk factor for incisional hernia and wound dehiscence [33]. In opposition to the above cited studies and in accordance with our study Williams et al. [28] reported in their study that no significant difference in body mass index and resident level was found when comparing SL:WL ratios above or equal and below 4:1.

In our findings, the mean stitch length was 1.5 cm in dogs and 1.1 cm in cats. In humans, a mean stitch length greater than 4 cm generated a three times higher rate of incisional hernia than a mean stitch length less than 4 cm. Additionally, the rate of wound infection doubled with each centimeter increase in stitch length [8]. In another prospective clinical study, a longer stitch was described to be associated with a higher wound infection rate [34]. Overall, although the mean SL:WL ratio in our study was less than the recommended 4:1 value, the mean stitch length was small, which is in accordance with current recommendations [6–8,34].

Current small animal surgical textbooks recommend TBs of 3 to 10 mm and SIs of 3 to 12 mm, depending on the animal’s size [16,21–24]. Only one book chapter mentioned specific recommendations for cats [20].

However, although SL:WL ratio is mentioned in a current small animal surgery textbook [23], neither the relationship between the TB and SI nor the stitch length are described anywhere in the small animal veterinary literature. For instance, taking the 2 lowest values of the textbook recommendations, i.e., a 3-mm TB combined with a 3-mm SI, will result in an SL:WL ratio of 4.1:1. However, a 3-mm TB combined with a 12-mm SI will result in an SL:WL ratio of 1.4:1. This finding is in contrast to human medicine suggestions for midline abdominal incision closure where a TB of 5–8 mm and an SI of every 5 mm are usually recommended [2,6,35]. Using these recommendations, an SI equal to the minimum TB value will always result in an SL:WL ratio greater than 4:1. It must be clearly noted that textbooks suggest to use TBs and SIs depending on the animal´s size [16,21–24]. Our results also showed a positive correlation between mean SI and mean TB values and weight categories.

Despite the low SL:WL ratio observed in our study, our results matched Rosin`s textbook recommendations [21] for TBs and SIs in 75 dogs. Within the SL:WL ratio of 3:1-<5:1, 27 dogs matched the TB and SI recommended in Fossum`s textbook [22]. The TB and SI in 21 cats with an SL:WL ratio ranging from 3:1 to <5:1 agreed with the feline textbook recommendations [20], as shown in Table 6. These findings suggest that applying the current textbook recommendations without specifying the TB to SI ratio cannot help surgeons reach the recommended 4:1 ratio. When comparing the model of the abdominal wall of an artificial medium-sized dog used in the in vitro study by Klonner [19] with our study, the values for TB and SI leading to an SL:WL ratio between 2:1-<4:1 were similar. Klonner [19] found a mean TB ranging between 4.1 and 5.1 mm and a mean SI ranging between 6.1 and 6.0 mm. In our study, the corresponding mean TB values were 3.2 to 4.6 mm, and the mean SI values were 6.1 to 6.4 mm. This finding could suggest that the surgical technique that led to an SL:WL ratio less than 4:1 was similar in both the in vitro and in vivo settings.

In our study, the SL and WL were measured, the number of stitches was counted, and all other suture parameters and ratios were calculated. Statistical analysis showed no correlations between the SL:WL ratio and the SI, whereas a moderate negative correlation was found between the SL:WL ratio and the mean SI:TB ratio. In our study, closure was performed with a mean TB of 3.2 mm and a mean SI of 6.2 mm in dogs and of 2.2 mm and 4.2 mm in cats. This resulted in a mean SI:TB ratio between 2.4 and 3.2, which cannot lead to an SL:WL ratio close to 4:1. In fact, following Jenkins’s [12] model and Klonner’s study [19], the SI should be equal to the TB to reach an SL:WL ratio close to or greater than 4:1. Therefore, the SI:TB ratio was proposed by Klonner [19] as a tool to predict the intended SL:WL ratio during surgery. With a given TB, the SL:WL ratio increases when the SI decreases; or at a given SI, the SL:WL ratio increases when the TB increases. Hassan et al. [18] reported that with a stable TB and an increase in the SI, the SL:WL ratio decreased. In our study, (see Table 6), the mean SI was between 6.0 and 6.4 mm in dogs and 3.7 and 4.8 mm in cats. These findings clearly demonstrate that increasing the TB with a constant SI results in a higher SL:WL ratio. However, the strategy to achieve higher SL:WL ratios by increasing the TB is limited, in a study [36] looking at abdominal closure in people with a BMI of 24 (normal weight), 5 mm tissue bites and stitch interval led to less incisional hernias than 10 mm tissue bites and stitch interval.

The majority of all closures was within an SL:WL ratio of 2:1-<3:1. Only a minimal number of closures achieved the recommended SL:WL ratio of 4:1 or higher although some participants were aware of the SL:WL ratio concept. These results suggest that previous knowledge of the SL:WL ratio principle had no influence on the way closure was achieved.

No incisional hernia formation was found in this study. In large animals, the incidence of hernia formation was greater than 16% in one study [37]. Although this number seems to be far less in small animals, only sparse scientific literature exists [23]. Berzon [38] noted incisional hernia rates of 0.8% in dogs and cats that underwent elective ovariohysterectomies performed by veterinary students under direct supervision. No incisional hernias were detected in a group of 70 cats, in which abdominal closure was performed in a continuous pattern using polydioxanone suture material [39].

### Limitations of this study

There are several limitations to this study.

First, although atraumatic surgery rules and appositional closure were recommended, the true tension on the suture material was not evaluated. It can be hypothesized that the more the surgeon pulls on the suture, the more the wound edges will tend to “collapse”, and the smaller the calculated tissue bites will be. This could lead to a smaller SL:WL ratio.

Not only the SL:WL ratio influences hernia formation [9,10,12–14], but there is experimental evidence that closure with a high SL:WL ratio (≥4:1) performed with low suture tension results in significantly better ultrastructural composition of the regenerating tissue (increase in the mean collagen protein concentration and tensile strength of the regenerating tissue) and leads to a significantly positive impact on the mechanical strength of the incision [40,41]. Höer et al. [42] reported that a loose fascial closure in rats with a continuous pattern performed with a distance holder of 2.4–4.8 mm showed no hernia formation or dehiscence after 28 days. However, when using a distance holder of 7.2 mm, incisional hernias developed in all animals within 28 days after operation. In a human study, a separation of 12 mm and more showed a 94.4% probability of herniation 1 month after operation [43].

In contrast, high suture tension leads to weakened regenerative tissue [41], and the percentage of collagen type III decreases in the incisional region [40]. Furthermore, high suture tension results in a 70% reduction in tissue perfusion [44].

In a porcine laparotomy model the suture tension dynamics with an implantable sensor device were measured. The mean tension of fascial closure showed a reduction of 24.3% after 30 minutes, and of almost 50% after 23 hours [45]. In conclusion these studies illustrate that both too low and too high suture tension are detrimental. Further studies evaluating the tension exerted on suture material could be beneficial.

The influence of different SL:WL ratios on short-term wound healing cannot be extrapolated from this study as only 29 patients were available for long-term follow-up. Given the very low rate of postoperative hernia in small abdominal closures [23,38,39,46], the influence of the SL:WL ratio on the incisional hernia rate was impossible. Also, the sole clinical examination might not be sufficient to evaluate hernia formation. In a systemic review comparing physical examination and imaging (ultrasound and CT-scan) Kroese et al. [47] reported a disagreement ranging between 7.6% and 39% CT-scan was reported as the most precise diagnostic method.

Further studies including diagnostic imaging and histologic evaluation of abdominal wall closures performed with different SL:WL ratios should be evaluated.

## Conclusions

In this study, the mean SL:WL ratio for abdominal closure was 2.5 ± 0.7:1. No influence of the surgeons’ experience or of the patients’ body weights or BCSs on the SL:WL ratio could be found. Our results suggest that too large an SI in relation to the TB is the reason why the recommended SL:WL ratio of 4:1 could not be reached. Current textbook recommendations mention TBs and SIs but no TB:SI ratios; hence, no recommendation on the SL:WL ratio can be extrapolated. In the future, clear recommendations on the TB:SI ratio would help surgeons reach the recommended SL:WL ratio > 4:1.

## Supporting information

S1 Table Descriptive data of 175 animals classified as all patients, dogs and cats: Mean age ± standard deviation (SD), mean body weight ± SD and mean body condition score (BCS) ± SD. (PDF)

S2 Table Frequency of distribution according breeds. (PDF)

S3 Table Frequency of distribution according procedures performed. (PDF)

S1 Data Mathematical approach to the calculation of TB. (PDF)

S2 Data (PDF)

## Abbreviations

BCS: body condition score
ECAR: European College of Animal Reproduction
ECVS: European College of Veterinary Surgery
mean SI: mean suture interval
mean SI:TB ratio: mean stitch interval to mean tissue bite ratio
mean STL: mean stitch length
mean TB: mean tissue bite
SD: standard deviation
SL: suture length
SL:WL ratio: suture length to wound length ratio
USP: United States Pharmacopeia
WL: wound length
