# Supplementary material for: Suture length to wound length ratio in 175 small animal abdominal midline closures
Source: PLoS One. 2019 May 20;14(5):e0216943. doi: 10.1371/journal.pone.0216943 (PMC6527205; doi:10.1371/journal.pone.0216943)
Supplement: S1 Table — (PDF) [file pone.0216943.s001.pdf]

	**All**	**Dogs**	**Cats**
	175	100	75
Mean age ± SD [year]	5.85 ± 4.4	6.56 ± 4.2	4.93 ± 4.4
Mean age ± SD [month]	70.23 ± 52.5	78.77 ± 50.7	59.11 ± 53.1
Mean body weight ± SD [kg]	12.37 ± 12.9	19.04 ± 13.8	3.67 ± 1.1
Mean BCS ± SD	4.91 ± 0.9	5.05 ± 0.9	4.72 ± 0.9
BCS 1	0	0	0
BCS 2	1 (0.6%)	0	1 (1.3%)
BCS 3	11 (6.3%)	5 (5%)	6 (8%)
BCS 4	37 (21.1%)	18 (18%)	19 (25.3%)
BCS 5	87 (49.7%)	50 (50%)	37 (49.3%)
BCS 6	33 (18.9%)	22 (22%)	11 (14.7%)
BCS 7	5 (2.9%)	4 (4%)	1 (1.3%)
BCS 8	1 (0.6%)	1 (1%)	0
BCS 9	0	0	0
