# Supplementary material for: Suture length to wound length ratio in 175 small animal abdominal midline closures
Source: PLoS One. 2019 May 20;14(5):e0216943. doi: 10.1371/journal.pone.0216943 (PMC6527205; doi:10.1371/journal.pone.0216943)
Supplement: S2 Table — (PDF) [file pone.0216943.s002.pdf]

Number of dogs	Breed
1	Affenpinscher, Australian Shepherd, Basset Hound, Bolonka Zwetna, Boxer, Cane Corso Italiano, Collie, Curly Coated Retriever, Dachshund, Dalmatian dog, German Shorthaired Pointer, German Longhaired Pointer, Fox Terrier Wire, Fox Terrier Smooth, French Bulldog, Havanese dog, Irish Red Setter, Jack Russell Terrier, Vizsla, Pug, Newfoundland, Austrian Pinscher, Pekinese, Giant Schnauzer, Staffordshire Bullterrier, Tibetan Spaniel, Weimaraner Shorthaired
2	Bavarian Mountain Scenthound, Bernese Mountain Dog, Dachshund Smooth-haired, German Shepherd Dog, Transylvanian Hound, Papillon, Flat Coated Retriever, Golden Retriever, Rhodesian Ridgeback, Beagle
3	Yorkshire Terrier
4	American Staffordshire Terrier, American Cocker Spaniel, Labrador Retriever
5	Maltese
8	Chihuahua
25	Mixed breed
**Number of cats**	**Breed**
1	Bengale, Longhair, Siamese
4	British Short Haired, Maine Coon
64	European Shorthair
