# Supplementary material for: Suture length to wound length ratio in 175 small animal abdominal midline closures
Source: PLoS One. 2019 May 20;14(5):e0216943. doi: 10.1371/journal.pone.0216943 (PMC6527205; doi:10.1371/journal.pone.0216943)
Supplement: S3 Table — (PDF) [file pone.0216943.s003.pdf]

**Number of animals**	**Operation**
1	*adrenalectomy, cholecystotomy tube, intussusception, peritoneopericardial hernia, partial prostatectomy*
2	*diaphragmatic hernia, extrahepatic portosystemic shunt ligation, omentalization of pancreas and prostate, pancreatectomy, partial hepatic lobectomy, subcutaneous ureteral bypass*
3	*abdominal wall hernia, cholecystectomy, partial gastrectomy*
4	*gastropexy*
6	*caesarean section, colopexy*
11	*cystotomy, enterectomy, enterotomy*
13	*splenectomy*
18	*diagnostic laparotomy*
25	*ovariohysterectomy*
44	*ovariectomy*
