# Supplementary material for: Suture length to wound length ratio in 175 small animal abdominal midline closures
Source: PLoS One. 2019 May 20;14(5):e0216943. doi: 10.1371/journal.pone.0216943 (PMC6527205; doi:10.1371/journal.pone.0216943)
Supplement: S1 Data — (PDF) [file pone.0216943.s004.pdf]

$$c^2 = a^2 + b^2$$

$$STL = c + 2TB$$

$$c = STL - 2TB$$
$$(STL - 2TB)^2 = SI^2 + (2TB)^2$$
$$STL^2 - 2STL2TB + (2TB)^2 = SI^2 + (2TB)^2$$
$$STL^2 - 2STL2TB = SI^2$$
$$STL^2 - SI^2 = 2STL2TB$$

$$TB = \frac{STL^2 - SI^2}{4STL}$$
