# Supplementary material for: Suture length to wound length ratio in 175 small animal abdominal midline closures
Source: PLoS One. 2019 May 20;14(5):e0216943. doi: 10.1371/journal.pone.0216943 (PMC6527205; doi:10.1371/journal.pone.0216943)
Supplement: S2 Data — (PDF) [file pone.0216943.s005.pdf]

number	surgeon	species	breed	body_weigth_kg
1	Resident	D	Chihuahua	2.7
2	Diplomate	D	German Shorthaired Pointer	32
3	Resident	D	American Cocker Spaniel	17.6
4	Resident	C	British Short Haired	3.9
5	Resident	D	Bernese Mountain Dog	50.2
6	Diplomate	C	European Shorthair	4.3
7	Diplomate	C	European Shorthair	3.5
8	Resident	D	Pug	8.2
9	Diplomate	D	Fox Terrier Smooth	10.1
10	Diplomate	C	European Shorthair	4.7
11	Resident	D	Beagle	10.0
12	Resident	C	British Short Haired	4
13	Resident	C	European Shorthair	2.5
14	Resident	C	European Shorthair	4
15	Resident	D	Maltese	2.4
16	Resident	D	Bernese Mountain Dog	40
17	Resident	D	Yorkshire Terrier	6.5
18	Diplomate	D	Rhodesian Ridgeback	40.5
19	Resident	C	European Shorthair	2.7
20	Resident	C	British Short Haired	4.8
21	Resident	C	European Shorthair	3.4
22	Resident	D	Vizsla	17.2
23	Diplomate	D	Mixed breed	22
24	Resident	D	Labrador Retriever	28.6
25	Resident	D	German Shepherd Dog	25
26	Resident	D	Papillon	3.6
27	Resident	C	European Shorthair	3.5
28	Resident	C	European Shorthair	3.4
29	Resident	D	Mixed breed	4
30	Diplomate	D	Cane Corso Italiano	31.1
31	Diplomate	D	American Staffordshire Terrier	26
32	Diplomate	C	European Shorthair	2.7
33	Resident	D	Chihuahua	2.7
34	Resident	C	Siamese	3.5
35	Resident	D	Mixed breed	5.4
36	Resident	C	European Shorthair	3
37	Diplomate	C	European Shorthair	2.7
38	Resident	D	Bolonka Zwetna	8.6
39	Resident	D	Mixed breed	9.7
40	Resident	C	European Shorthair	5.5
41	Diplomate	D	Labrador Retriever	26.7
42	Resident	D	Transylvanian Hound	42.3
43	Resident	C	European Shorthair	3.1
44	Resident	C	European Shorthair	3.8
45	Diplomate	D	Australian Shepherd	31.2
46	Resident	C	European Shorthair	2.5
47	Resident	D	Mixed breed	38.8
48	Diplomate	D	Rhodesian Ridgeback	24
49	Resident	D	American Cocker Spaniel	14

50	Diplomate	D	Labrador Retriever	19
51	Diplomate	C	European Shorthair	2.5
52	Diplomate	C	European Shorthair	2.6
53	Diplomate	D	Irish Red Setter	27.5
54	Resident	D	Mixed breed	5.7
55	Resident	D	American Staffordshire Terrier	25.6
56	Diplomate	C	European Shorthair	2.1
57	Diplomate	D	American Cocker Spaniel	16
58	Diplomate	D	Chihuahua	2
59	Diplomate	D	Mixed breed	25
60	Diplomate	C	European Shorthair	3.6
61	Resident	C	European Shorthair	4.8
62	Resident	D	American Staffordshire Terrier	25.5
63	Resident	D	Mixed breed	12.5
64	Resident	D	Mixed breed	30
65	Diplomate	C	European Shorthair	2.8
66	Resident	D	Collie	18
67	Diplomate	D	Chihuahua	5.4
68	Resident	D	Transylvanian Hound	41.5
69	Diplomate	D	Tibetan Spaniel	4.5
70	Resident	D	Chihuahua	2.7
71	Diplomate	D	Yorkshire Terrier	3.8
72	Diplomate	D	French Bulldog	10.3
73	Diplomate	D	Papillon	5.8
74	Diplomate	D	European Shorthair	3
75	Resident	C	European Shorthair	4.7
76	Diplomate	D	Mixed breed	12.8
77	Diplomate	C	European Shorthair	4
78	Diplomate	D	Affenpinscher	5.7
79	Diplomate	D	Mixed breed	15.8
80	Diplomate	D	Fox Terrier Wire	8.4
81	Diplomate	C	European Shorthair	4.1
82	Diplomate	C	European Shorthair	2.9
83	Diplomate	C	European Shorthair	2.4
84	Diplomate	C	European Shorthair	3
85	Diplomate	C	Maine Coon	6.7
86	Resident	D	Weimaraner Shorthaired	51
87	Diplomate	C	European Shorthair	3.6
88	Resident	D	Mixed breed	14.1
89	Diplomate	D	Yorkshire Terrier	3.4
90	Diplomate	C	European Shorthair	3.8
91	Resident	D	Maltese	6.7
92	Diplomate	D	Mixed breed	26.3
93	Diplomate	C	Longhair	3.5
94	Resident	D	Staffordshire Bullterrier	18.2
95	Resident	D	Bavarian Mountain Scenthound	15.7
96	Resident	D	Mixed breed	20
97	Resident	C	European Shorthair	3
98	Resident	C	European Shorthair	3.2
99	Resident	C	European Shorthair	3

100	Resident	C	European Shorthair	2.9
101	Resident	D	Mixed breed	9.6
102	Resident	D	Newfoundland	75
103	Diplomate	D	German Shepherd Dog	31.2
104	Resident	D	Pekinese, Pekingese	7.5
105	Diplomate	D	Dachshund Smooth-haired	7.3
106	Resident	D	Mixed breed	14.6
107	Resident	D	Dalmatian dog	37.1
108	Resident	D	Chihuahua	2.9
109	Resident	C	European Shorthair	3.7
110	Diplomate	D	Mixed breed	11.1
111	Resident	D	Chihuahua	6
112	Diplomate	D	Mixed breed	44.6
113	Diplomate	C	European Shorthair	3.8
114	Resident	D	Mixed breed	13.7
115	Resident	D	American Staffordshire Terrier	25.5
116	Diplomate	C	British Short Haired	5.6
117	Diplomate	D	Basset Hound	35.1
118	Resident	D	Golden Retriever	31.5
119	Diplomate	D	Curly Coated Retriever	25
120	Diplomate	C	Bengale	4.9
121	Diplomate	D	Mixed breed	24.8
122	Resident	D	Flat Coated Retriever	32
123	Resident	C	European Shorthair	3.4
124	Resident	C	European Shorthair	3.8
125	Resident	C	Maine Coon	7.9
126	Diplomate	D	Mixed breed	13
127	Diplomate	D	Dachshund Smooth-haired	10.6
128	Diplomate	D	Havanese dog	4
129	Resident	C	European Shorthair	3.4
130	Resident	C	Maine Coon	8
131	Resident	C	European Shorthair	2.8
132	Resident	D	Labrador Retriever	36.8
133	Diplomate	D	Giant Schnauzer	10.8
134	Diplomate	C	European Shorthair	3.9
135	Resident	D	Mixed breed	37
136	Diplomate	D	Jack Russell Terrier	5.8
137	Diplomate	D	Bavarian Mountain Scenthound	23.8
138	Diplomate	C	European Shorthair	3.1
139	Resident	C	European Shorthair	4.1
140	Resident	C	Maine Coon	5.8
141	Resident	C	European Shorthair	2.8
142	Diplomate	D	Mixed breed	22.5
143	Diplomate	C	European Shorthair	3.2
144	Diplomate	C	European Shorthair	4.2
145	Diplomate	C	European Shorthair	3.4
146	Diplomate	C	European Shorthair	5.1
147	Diplomate	D	Flat Coated Retriever	30.5
148	Diplomate	D	American Cocker Spaniel	9.9
149	Diplomate	D	Boxer	25

150	Diplomate	D	Beagle	14.5
151	Diplomate	C	European Shorthair	3.8
152	Diplomate	C	European Shorthair	3.1
153	Diplomate	C	European Shorthair	2.5
154	Diplomate	C	European Shorthair	2.7
155	Diplomate	C	European Shorthair	3
156	Diplomate	C	European Shorthair	4
157	Diplomate	D	Austrian Pinscher	20
158	Diplomate	D	Maltese	3.2
159	Diplomate	C	European Shorthair	3.7
160	Diplomate	D	Golden Retriever	25.1
161	Diplomate	D	Maltese	4
162	Diplomate	D	Mixed breed	12.4
163	Diplomate	C	European Shorthair	3.7
164	Diplomate	C	European Shorthair	3.3
165	Diplomate	C	European Shorthair	3.4
166	Diplomate	D	Mixed breed	29.9
167	Diplomate	D	Dachshund	9.5
168	Diplomate	D	Maltese	4.6
169	Diplomate	C	European Shorthair	3.1
170	Diplomate	C	European Shorthair	4
171	Diplomate	C	European Shorthair	2.4
172	Diplomate	C	European Shorthair	3.5
173	Resident	C	European Shorthair	3.3
174	Resident	C	European Shorthair	2.9
175	Diplomate	D	German Longhaired Pointer	37.5

BCS_1-9	gender	year	month	SL_after_positioning_the_first_knot_cm
5	m	8	1	74.7
3	m	3	8	83
6	nm	12	7	70.3
5	nm	2	5	72.5
4	nm	7	1	87.9
6	sf	9	0	73
4	nm	11	8	73.5
5	m	2	11	75.6
4	m	0	10	86.5
5	nm	5	6	75
5	sf	7	11	72.1
6	f	3	1	68.5
5	f	0	6	68.8
5	f	3	2	68
5	f	7	5	67.5
5	m	10	3	67.8
5	m	8	10	75.2
6	f	7	7	69.2
5	f	0	7	69
6	f	4	4	66.2
5	f	6	6	65.3
3	f	1	4	83.5
6	sf	1	11	81.1
6	f	8	7	81
4	m	0	10	84.3
5	m	2	6	73
6	f	2	6	26
5	f	2	6	73.7
5	f	1	0	56.4
4	f	4	9	82.2
5	f	2	6	82
4	f	1	1	77
5	sf	4	1	74.7
5	f	1	4	70.5
5	f	7	6	71.1
4	f	3	3	71.4
3	nm	5	3	74.1
5	sf	8	0	85
5	f	1	4	70.4
6	nm	3	10	74
5	nm	9	7	70
6	f	10	9	66.8
5	f	0	11	68.5
5	f	3	5	71.5
7	m	10	8	68
5	f	0	6	70.3
7	f	4	0	84
5	f	0	8	80.8
5	f	7	6	78.9

5	f	1	3	86
4	f	0	10	74.7
4	f	0	10	77
5	f	9	7	86.4
3	nm	2	7	35.8
5	f	1	1	77.8
2	sf	12	7	75
5	f	8	0	85.5
4	sf	7	1	75
5	f	7	1	70.5
4	sf	6	10	75
6	sf	10	0	77.6
6	f	10	11	88.6
4	m	8	8	86.8
5	nm	10	10	80
3	nm	17	0	74.2
5	f	6	5	70.5
5	m	0	7	72.4
6	f	10	10	71
4	f	3	2	75.3
5	f	1	1	71.3
6	f	10	5	74.7
5	sf	9	7	74
6	m	3	8	73.4
5	f	0	9	74.7
6	nm	2	8	59.6
5	f	2	0	72.5
5	sf	14	6	73.8
5	f	7	1	71
4	f	1	1	70.5
5	f	7	4	73.5
5	f	13	9	76.5
5	f	0	10	76
4	f	0	10	74
5	f	0	10	76
4	sf	9	3	74
8	m	6	8	75
4	nm	1	6	74.1
6	sf	9	11	80.7
5	sf	10	6	70
5	sf	13	9	72.9
5	f	4	2	72.6
6	sf	2	3	82.5
4	f	1	1	76.9
5	f	3	0	73
4	f	1	1	87
6	f	1	0	83.4
5	f	5	4	75.8
5	f	5	3	30.2
4	f	8	0	36.5

5	f	0	6	68.5
5	f	0	9	74.3
7	f	3	4	83.4
3	sf	10	6	85.3
6	nm	13	5	84.7
5	f	7	4	77
6	sf	14	8	72
7	nm	5	8	90
5	m	7	5	69
4	nm	13	0	69.2
4	m	0	10	72.3
6	m	8	6	73
5	m	12	2	86
4	sf	5	11	74.2
4	nm	14	3	75.2
5	m	10	1	85
7	nm	5	8	75.4
4	m	5	4	86
4	m	1	3	79
4	sf	6	1	83.3
5	nm	8	4	78.5
4	f	2	4	74.4
6	sf	13	9	73
4	sf	2	4	76.8
3	sf	10	6	77
5	nm	2	6	75.9
5	m	9	3	86.1
5	sf	9	9	72.3
5	sf	12	8	75.2
3	sf	8	4	78
5	nm	6	4	77
3	sf	11	2	77
6	m	12	8	68.5
5	m	0	2	77.7
4	nm	11	0	77.7
6	sf	13	10	84
5	sf	1	4	79
5	m	11	6	86
5	f	1	2	77
4	sf	5	5	77.5
4	nm	3	1	76
5	m	0	5	73.8
5	sf	10	4	84.5
5	nm	12	0	77
6	nm	2	0	76
5	m	6	0	67
6	nm	9	0	74.9
6	f	7	7	83.8
4	f	15	2	58.5
4	m	2	10	87

6	f	6	0	77.7
6	f	4	2	75.1
5	f	1	1	74.8
5	f	1	1	77.9
4	f	1	4	75.2
5	f	0	6	76.6
5	f	4	0	77
6	m	10	10	80.9
5	m	10	3	80.3
5	f	1	2	76.5
3	f	14	3	89
5	sf	1	10	79
6	m	8	11	79.2
5	nm	4	0	76
6	f	1	6	73.6
5	sf	6	1	80
5	sf	9	10	90.5
4	f	4	9	75.6
5	f	1	4	75.2
5	f	0	6	77.4
5	f	0	7	77.4
4	f	0	7	78.2
5	f	0	6	77.5
5	sf	12	10	77
3	sf	12	0	75.6
5	f	3	6	85.9

SL_before_positioning_the_last_knot_cm	SL_cm	WL_cm	SL_WL_ratio	number_of_stitches
59.5	15.2	7.4	2.1	12
35.4	47.6	19.0	2.5	27
40.6	29.7	14.7	2.0	18
41.7	30.8	10.0	3.1	20
41.4	46.5	23.0	2.0	26
56.5	16.5	8.0	2.1	15
46	27.5	9.0	3.1	15
64.5	11.1	4.9	2.3	11
56	30.5	9.0	3.4	14
53.4	21.6	9.5	2.3	16
30.9	41.2	9.6	4.3	23
61.6	6.9	3.4	2.0	10
62.4	6.4	2.8	2.3	9
59.7	8.3	2.8	3.0	9
56	11.5	5.3	2.2	17
19.1	48.7	17.0	2.9	23
64	11.2	6.5	1.7	12
18.2	51	22.5	2.3	36
61.6	7.4	3.1	2.4	9
49.1	17.1	6.4	2.7	20
56	9.3	2.6	3.6	10
60.5	23	9.6	2.4	20
25	56.1	22.5	2.5	27
44.5	36.5	15.3	2.4	29
43.6	40.7	15.5	2.6	19
54	19	5.6	3.4	13
23.2	2.8	2.0	1.4	6
69.9	3.8	2.5	1.5	6
47.4	9	5.0	1.8	10
63.7	18.5	9.3	2.0	14
55.8	26.2	13.0	2.0	22
70.5	6.5	2.3	2.8	10
65.7	9	3.0	3.0	9
57	13.5	7.2	1.9	21
63.4	7.7	5.3	1.5	10
61.9	9.5	2.4	4.0	9
54.1	20	8.5	2.4	15
39.4	45.6	15.4	3.0	27
62	8.4	7.0	1.2	10
39	35	11.0	3.2	24
16	54	23.0	2.3	22
19.7	47.1	18.0	2.6	16
59.6	8.9	2.1	4.2	11
61.1	10.4	2.6	4.0	10
24	44	20.3	2.2	24
61.9	8.4	2.8	3.0	9
52.5	31.5	17.0	1.9	18
59.4	21.4	11.0	1.9	19
56.7	22.2	8.8	2.5	18

73.3	12.7	7.5	1.7	15
70	4.7	2.3	2.0	9
69	8	3.0	2.7	10
62	24.4	13.5	1.8	20
25.6	10.2	3.4	3.0	7
60.8	17	6.5	2.6	13
47	28	13.0	2.2	18
61.3	24.2	11.0	2.2	19
53.1	21.9	9.5	2.3	18
28	42.5	16.0	2.7	15
40	35	11.0	3.2	19
64.6	13	6.5	2.0	14
57.1	31.5	17.4	1.8	21
35.8	51	15.3	3.3	21
52.3	27.7	8.5	3.3	12
54	20.2	10.8	1.9	19
47.3	23.2	14.0	1.7	23
33	39.4	11.0	3.6	23
43.6	27.4	11.7	2.3	17
65.5	9.8	4.0	2.5	14
62.2	9.1	4.8	1.9	15
52.5	22.2	8.5	2.6	32
44	30	11.5	2.6	17
48.1	25.3	10.0	2.5	20
67.5	7.2	3.0	2.4	12
27.4	32.2	11.0	2.9	20
62.5	10	4.0	2.5	13
15.3	58.5	13.0	4.5	34
57.5	13.5	4.2	3.2	18
61.5	9	4.5	2.0	11
53	20.5	8.0	2.6	22
70	6.5	3.5	1.9	13
68.5	7.5	3.0	2.5	13
67.5	6.5	3.0	2.2	12
69.5	6.5	2.6	2.5	12
54	20	7.8	2.6	13
21.6	53.4	16.0	3.3	19
57	17.1	9.5	1.8	15
26.3	54.4	17.5	3.1	22
38.4	31.6	10.4	3.0	21
42.4	30.5	9.0	3.4	16
51.5	21.1	8.4	2.5	26
31.3	51.2	19.2	2.7	22
60	16.9	7.0	2.4	21
46.8	26.2	10.9	2.4	29
75	12	5.7	2.1	13
69.9	13.5	6.0	2.3	14
73.9	1.9	1.8	1.1	8
27.6	2.6	1.8	1.4	8
34	2.5	1.6	1.6	6

65	3.5	3.0	1.2	10
64.6	9.7	5.0	1.9	17
40.1	43.3	16.5	2.6	26
17.2	68.1	23.5	2.9	39
67.5	17.2	9.5	1.8	13
41	36	13.5	2.7	24
36	36	16.0	2.3	20
70	20	7.0	2.9	10
55.6	13.4	3.8	3.5	8
40.9	28.3	9.5	3.0	15
48	24.3	13.3	1.8	18
64	9	4.8	1.9	9
27	59	26.0	2.3	21
55.5	18.7	10.3	1.8	17
31.7	43.5	23.1	1.9	30
42	43	15.6	2.8	17
46.7	28.7	12.8	2.2	20
48.5	37.5	16.0	2.3	22
39.9	39.1	16.1	2.4	18
29.8	53.5	25.0	2.1	30
68.3	10.2	6.0	1.7	13
54.6	19.8	13.5	1.5	22
35	38	12.0	3.2	16
52.6	24.2	9.8	2.5	19
54	23	11.0	2.1	17
50.5	25.4	10.4	2.4	21
52	34.1	11.2	3.0	11
24	48.3	16.0	3.0	19
41.4	33.8	10.9	3.1	15
42	36	10.5	3.4	22
27.5	49.5	13.0	3.8	30
41.5	35.5	10.8	3.3	26
29.5	39	15.0	2.6	21
36.7	41	11.5	3.6	24
60	17.7	10.5	1.7	15
26	58	17.0	3.4	27
39	40	11.8	3.4	22
28.2	57.8	18.0	3.2	26
67.5	9.5	3.2	3.0	14
43	34.5	12.0	2.9	23
49.2	26.8	8.0	3.4	15
59.3	14.5	5.0	2.9	11
47.2	37.3	18.0	2.1	22
39	38	8.5	4.5	17
45.7	30.3	9.5	3.2	18
49	18	8.3	2.2	13
35.4	39.5	11.8	3.3	16
66	17.8	11.3	1.6	17
45.3	13.2	9.0	1.5	17
32.5	54.5	16.0	3.4	23

64.5	13.2	9.4	1.4	16
67.2	7.9	4.8	1.6	11
71.5	3.3	2.5	1.3	8
72.6	5.3	2.8	1.9	9
69.3	5.9	3.6	1.6	10
71.9	4.7	2.4	2.0	7
72.5	4.5	3.0	1.5	10
56	24.9	8.0	3.1	10
50.5	29.8	8.3	3.6	18
72.8	3.7	2.2	1.7	7
71.3	17.7	9.1	1.9	17
32.5	46.5	13.3	3.5	29
33	46.2	12.5	3.7	27
32	44	12.6	3.5	27
45	28.6	8.7	3.3	19
45.7	34.3	9.3	3.7	19
28	62.5	18.0	3.5	25
62.3	13.3	11.2	1.2	21
65.1	10.1	6.5	1.6	20
74.3	3.1	2.2	1.4	7
74.1	3.3	2.2	1.5	8
75.2	3	2.2	1.4	8
74.3	3.2	2.7	1.2	8
54	23	9.5	2.4	17
51.3	24.3	12.0	2.0	19
66.1	19.8	14.8	1.3	17

mean_ STL_cm	mean_SI_cm	mean_ TB_cm	SI_TB_Ratio	USP	complication	Follow_up
1.27	0.62	0.24	2.55	2	n	y
1.76	0.70	0.37	1.90	0	n	y
1.65	0.82	0.31	2.62	0	n	n
1.54	0.50	0.34	1.45	2	n	n
1.79	0.88	0.34	2.62	0	n	n
1.10	0.53	0.21	2.54	2	n	n
1.83	0.60	0.41	1.47	2	n	y
1.01	0.45	0.20	2.19	2	n	y
2.18	0.64	0.50	1.29	0	n	n
1.35	0.59	0.27	2.18	2	n	y
1.79	0.42	0.42	0.99	2	n	n
0.69	0.34	0.13	2.60	3	n	n
0.71	0.31	0.14	2.16	2	n	n
0.92	0.31	0.20	1.52	2	n	n
0.68	0.31	0.13	2.34	3	n	n
2.12	0.74	0.46	1.59	1	n	n
0.93	0.54	0.15	3.50	2	n	n
1.42	0.63	0.29	2.19	1	n	n
0.82	0.34	0.17	2.03	2	n	n
0.86	0.32	0.18	1.74	2	n	n
0.93	0.26	0.21	1.21	2	n	n
1.15	0.48	0.24	2.02	0	n	n
2.08	0.83	0.44	1.91	0	n	y
1.26	0.53	0.26	2.03	0	n	n
2.14	0.82	0.46	1.78	0	n	n
1.46	0.43	0.33	1.29	2	n	n
0.47	0.33	0.06	5.83	2	n	n
0.63	0.42	0.09	4.64	2	n	n
0.90	0.50	0.16	3.21	2	n	n
1.32	0.66	0.25	2.69	0	n	n
1.19	0.59	0.22	2.63	0	n	n
0.65	0.23	0.14	1.62	3	n	n
1.00	0.33	0.22	1.50	2	n	n
0.64	0.34	0.12	2.98	3	n	n
0.77	0.53	0.10	5.23	2	y	y
1.06	0.27	0.25	1.08	3	n	n
1.33	0.57	0.27	2.07	2	n	n
1.69	0.57	0.37	1.52	0	n	n
0.84	0.70	0.06	10.91	2	n	n
1.46	0.46	0.33	1.39	2	n	n
2.45	1.05	0.50	2.08	1	n	n
2.94	1.13	0.63	1.79	1	n	y
0.81	0.19	0.19	1.00	3	n	n
1.04	0.26	0.24	1.07	3	n	n
1.83	0.85	0.36	2.34	1	n	y
0.93	0.31	0.21	1.50	3	n	n
1.75	0.94	0.31	3.05	1	n	n
1.13	0.58	0.21	2.79	0	n	n
1.23	0.49	0.26	1.88	0	n	n

0.85	0.50	0.14	3.63	0 n	n
0.52	0.26	0.10	2.57	3 n	n
0.80	0.30	0.17	1.75	3 n	n
1.22	0.68	0.21	3.19	0 n	n
1.46	0.49	0.32	1.50	2 n	n
1.31	0.50	0.28	1.79	0 n	n
1.56	0.72	0.31	2.37	2 n	n
1.27	0.58	0.25	2.29	0 n	y
1.22	0.53	0.25	2.14	2 n	n
2.83	1.07	0.61	1.75	1 n	n
1.84	0.58	0.42	1.39	2 n	y
0.93	0.46	0.17	2.67	2 n	n
1.50	0.83	0.26	3.18	0 n	n
2.43	0.73	0.55	1.32	0 n	n
2.31	0.71	0.52	1.36	0 n	n
1.06	0.57	0.19	2.99	2 n	n
1.01	0.61	0.16	3.80	2 n	n
1.71	0.48	0.39	1.21	2 n	n
1.61	0.69	0.33	2.09	1 n	n
0.70	0.29	0.15	1.96	2 n	n
0.61	0.32	0.11	2.92	3 n	y
0.69	0.27	0.15	1.79	3 n	n
1.76	0.68	0.38	1.80	2 n	n
1.27	0.50	0.27	1.87	2 n	n
0.60	0.25	0.12	2.02	2 n	n
1.61	0.55	0.36	1.55	2 n	n
0.77	0.31	0.16	1.90	2 n	n
1.72	0.38	0.41	0.94	2 n	n
0.75	0.23	0.17	1.38	2 n	n
0.82	0.41	0.15	2.67	2 n	n
0.93	0.36	0.20	1.84	3 n	n
0.50	0.27	0.09	3.03	3 n	n
0.58	0.23	0.12	1.90	3 n	n
0.54	0.25	0.11	2.35	3 n	n
0.54	0.22	0.11	1.90	3 n	n
1.54	0.60	0.33	1.84	2 n	n
2.81	0.84	0.64	1.32	1 n	n
1.14	0.63	0.20	3.21	2 n	n
2.47	0.80	0.55	1.44	0 n	n
1.50	0.50	0.34	1.48	2 n	n
1.91	0.56	0.44	1.29	2 n	n
0.81	0.32	0.17	1.89	2 n	n
2.33	0.87	0.50	1.75	0 n	n
0.80	0.33	0.17	2.00	3 n	n
0.90	0.38	0.19	2.01	2 n	n
0.92	0.44	0.18	2.45	0 n	n
0.96	0.43	0.19	2.22	0 n	n
0.24	0.23	0.01	36.97	3 n	n
0.33	0.23	0.04	5.32	2 n	n
0.42	0.27	0.06	4.34	2 n	n

0.35	0.30	0.02	12.92	3	n	n
0.57	0.29	0.10	2.81	2	n	n
1.67	0.63	0.36	1.78	0	n	n
1.75	0.60	0.38	1.57	0	n	n
1.32	0.73	0.23	3.18	0	n	n
1.50	0.56	0.32	1.75	2	n	n
1.80	0.80	0.36	2.22	2	n	n
2.00	0.70	0.44	1.60	0	n	n
1.68	0.48	0.39	1.23	2	n	n
1.89	0.63	0.42	1.51	2	n	y
1.35	0.74	0.24	3.13	2	n	n
1.00	0.53	0.18	2.98	2	n	y
2.81	1.24	0.57	2.19	0	n	y
1.10	0.61	0.19	3.16	2	n	y
1.45	0.77	0.26	2.96	2	n	n
2.53	0.92	0.55	1.67	0	n	n
1.44	0.64	0.29	2.23	2	n	y
1.70	0.73	0.35	2.09	0	n	n
2.17	0.89	0.45	1.98	0	n	n
1.78	0.83	0.35	2.39	0	n	y
0.78	0.46	0.13	3.60	2	n	n
0.90	0.61	0.12	5.10	2	n	n
2.38	0.75	0.53	1.40	1	n	y
1.27	0.52	0.27	1.94	2	n	n
1.35	0.65	0.26	2.48	2	n	n
1.21	0.50	0.25	1.97	2	n	n
3.10	1.02	0.69	1.47	0	n	y
2.54	0.84	0.57	1.49	2	n	n
2.25	0.73	0.50	1.44	2	n	n
1.64	0.48	0.37	1.28	2	n	n
1.65	0.43	0.38	1.13	2	n	y
1.37	0.42	0.31	1.34	2	n	y
1.86	0.71	0.40	1.81	0	n	n
1.71	0.48	0.39	1.22	2	n	n
1.18	0.70	0.19	3.66	2	n	n
2.15	0.63	0.49	1.28	0	n	n
1.82	0.54	0.41	1.29	2	n	y
2.22	0.69	0.50	1.38	0	n	n
0.68	0.23	0.15	1.52	2	n	n
1.50	0.52	0.33	1.58	2	n	y
1.79	0.53	0.41	1.31	2	n	n
1.32	0.45	0.29	1.57	2	n	n
1.70	0.82	0.33	2.52	0	n	n
2.24	0.50	0.53	0.94	2	n	y
1.68	0.53	0.38	1.39	2	n	n
1.38	0.64	0.27	2.34	2	n	n
2.47	0.74	0.56	1.31	2	n	n
1.05	0.66	0.16	4.25	0	n	n
0.78	0.53	0.10	5.10	2	n	n
2.37	0.70	0.54	1.29	0	n	y

0.83	0.59	0.10	5.78	2	n	n
0.72	0.44	0.11	3.85	2	n	n
0.41	0.31	0.04	7.11	2	n	n
0.59	0.31	0.11	2.93	2	n	n
0.59	0.36	0.09	3.89	2	n	n
0.67	0.34	0.12	2.76	2	n	n
0.45	0.30	0.06	4.80	3	n	n
2.49	0.80	0.56	1.43	1	n	n
1.66	0.46	0.38	1.21	2	n	n
0.53	0.31	0.09	3.68	3	n	n
1.04	0.54	0.19	2.80	0	n	n
1.60	0.46	0.37	1.25	2	n	y
1.71	0.46	0.40	1.17	2	n	n
1.63	0.47	0.37	1.25	2	n	n
1.51	0.46	0.34	1.34	2	n	n
1.81	0.49	0.42	1.17	2	n	n
2.50	0.72	0.57	1.26	0	n	n
0.63	0.53	0.05	11.58	2	n	n
0.51	0.33	0.07	4.39	2	n	n
0.44	0.31	0.05	5.72	3	n	n
0.41	0.28	0.06	4.80	3	n	n
0.38	0.28	0.04	6.35	3	n	n
0.40	0.34	0.03	11.72	3	n	n
1.35	0.56	0.28	1.99	2	n	y
1.28	0.63	0.24	2.61	2	n	y
1.16	0.87	0.13	6.78	0	n	n
